# Do genetic and environmental influences on disordered eating change from early to late adolescence?

**DOI:** 10.1186/2050-2974-3-S1-O10

**Published:** 2015-11-23

**Authors:** A Kate Fairweather-Schmidt, Tracey D Wade

**Affiliations:** 1 Flinders University, Australia

## 

Genetic and environmental contributions to the global Eating Disorders Examination (EDE) scores over early and later adolescence were investigated to identify whether different sources influence disordered eating (DE). Specific sources of environmental risk were also examined for differences early and late in the adolescent developmental trajectory.

Adolescent females from the Australian Twin Registry were interviewed by telephone, including the EDE and impairment/risk-related self-report measures. Data were collected at 12-15 and 16-19 years (Wave 1: N=699, 351 pairs; Wave 3: N=499, 247 pairs).

Analyses involved bivariate Cholesky decomposition modelling of genetic and non-shared environmental influences. At 12-15 years, additive genetic and non-shared environmental sources significantly contributed to the DE phenotype, continuing to contribute at 16-19 years; additional independent additive genetic and non-shared environmental sources also conveyed risk at ages 16-19. Linear mixed models of environmental risk identified weight-related peer teasing in early-mid adolescence predicted DE later in adolescence.

A second risk period for DE onset appears in late adolescence. The predominance of family relationships attenuates among teenagers synchronously with a strong surge in attention to, and investment in, peer-peer connectivity. This accords with our findings implicating weight-related peer teasing - a non-shared environmental risk factor - in risk of DE. Increased genetic risk and sensitivity to non-shared environmental influences are likely antecedents in development of DE in late teenagehood.

